# Design and Construction of an ROV for Underwater Exploration

**DOI:** 10.3390/s19245387

**Published:** 2019-12-06

**Authors:** Oscar Adrian Aguirre-Castro, Everardo Inzunza-González, Enrique Efrén García-Guerrero, Esteban Tlelo-Cuautle, Oscar Roberto López-Bonilla, Jesús Everardo Olguín-Tiznado, José Ricardo Cárdenas-Valdez

**Affiliations:** 1 UABC, Faculty of Engineering, Architecture and Design, Autonomous University of Baja California, Ensenada 22860, Baja California, Mexico; oscar.aguirre@uabc.edu.mx (O.A.A.-C.); eegarcia@uabc.edu.mx (E.E.G.-G.); olopez@uabc.edu.mx (O.R.L.-B.); jeol79@uabc.edu.mx (J.E.O.-T.); 2 INAOE, Department of Electronics, Puebla 72840, Mexico; etlelo@inaoep.mx; 3 ITT, Department of Electrical and Electronic Engineering, Tijuana Institute of Technology, Tijuana 22500, Baja California, Mexico; jose.cardenas@tectijuana.edu.mx

**Keywords:** ROV, underwater exploration, video capture, subsea inspection and intervention, marine robotics, underwater technology, real time, vision, complementary filter, Raspberry Pi

## Abstract

The design of a remotely operated vehicle (ROV) with a size of 18.41 cm × 29.50 cm × 33.50 cm, and a weight of 15.64 kg, is introduced herein. The main goal is to capture underwater video by remote control communication in real time via Ethernet protocol. The ROV moves under the six brushless motors governed through a smart PID controller (Proportional + Integral + Derivative) and by using pulse-wide modulation with short pulses of 1 μs to improve the stability of the position in relation to the translational, ascent or descent, and rotational movements on three axes to capture images of 800 × 640 pixels on a video graphic array standard. The motion control, 3D position, temperature sensing, and video capture are performed at the same time, exploiting the four cores of the Raspberry Pi 3, using the threading library for parallel computing. In such a way, experimental results show that the video capture stage can process up to 42 frames per second on a Raspberry Pi 3. The remote control of the ROV is executed under a graphical user interface developed in Python, which is suitable for different operating systems, such as GNU/Linux, Windows, Android, and OS X. The proposed ROV can reach up to 100 m underwater, thus solving the issue of divers who can only reach 30 m depth. In addition, the proposed ROV can be useful in underwater applications such as surveillance, operations, maintenance, and measurement.

## 1. Introduction

Recently, several works on remotely operated vehicles (ROVs) have been reported for applications in ocean research [1,2,3,4]. In particular, ROVs have been used for underwater intervention, repair, and maintenance operations in offshore industries, including oil and gas industries, marine structures, marine sciences, naval defense, marine renewable energy, and scientific purposes [5,6,7,8,9]. Within submarine applications, recognition tasks stand out; for example, in [10,11,12] ROVs are employed for tracking mines and are programmed to carry out high-risk tasks, executing algorithms related to prediction, diagnosis, and classification. Other research efforts have been focused for underwater surveillance [13,14] and they are configured to manage continuous tasks with specific objectives. Some other research attempts have focused on the support in an optical fiber cable laying system on the seabed [15,16,17]. In [18,19], they are key to oceanographic research related to obtaining highly dynamic tidal data. In marine archeology, they are used for the exploration of underwater pools of toxic brine [20] and observation of underwater structures [21]. Regarding underwater communication, the authors of [22] proposed the use of a secure communication framework concealing the information under certain characteristics of pulses used by mammals. In addition, ROVs can be used to capture underwater images, of which nowadays there are open research lines [23,24,25,26]. However, despite the significant advances that have been achieved in the different areas of application and development of ROVs, it is a fertile field for research given the wide range of specialties that come into play in achieving optimal functionality. The precise sensing of underwater applications is a current topic in the research community. Efforts related to positioning a system based on GPS have been made; the characterization of the whole system in this case was done by means of a statistical study, considering different numbers of beacons [27]. Additionally, a novel programmable event-driven acoustic detector featuring audio pattern recognition for underwater deployment has been developed in [28]. Even a robust algorithm related to an autonomous underwater vehicle (AUV) was proposed to improve the supervised missions [29]. Currently, a real-time proposal of underwater acoustical imaging systems has been developed in [30], with the contribution of high-resolution underwater 3D acoustical imaging. Results based on implementation and experimental analysis of underwater stereo fusion and an algorithm for real-time 3D dense reconstruction with camera tracking  have been reported  [31]. In [32], the design and fabrication of an underwater remotely operated vehicle with single thruster configuration is reported. In [33], the design and implementation of an aquatic robot type ROV is described. The authors are focused on the design and implementation of an aquatic robot suitable for underwater exploration in order to improve its stability performance and achieving an efficient exploration system.

Nevertheless, aspects such as the ROV’s autonomy, efficiency in energy consumption, processing and information storage, hydrodynamic structure, position control, and limitations in communication are some of the topics on which current research could contribute [34,35,36,37]. Another factor that can affect the ROV design and implementation is the high cost involved, but it is important to consider an adequate balance between the cost and the functionality of the system in relation to its application [38,39,40,41]. On this issue, one can take advantage of embedded systems, which are very useful for solving real-world problems for different fields of application. For example, the System on a Chip (SoC) Raspberry Pi has been used to solve different problems of practical applications, such as the accounting of underwater fish [42] as special case in a fish farm, or to perform complex tasks such as parallel computing [43].

In this paper, we propose the use of an SoC Raspberry Pi 3 as an onboard computer, since it can be programmed by using open source software, it is cheap, it has multiprocessing capabilities, easy scheduling tasks, and the programming language Python can be used. In addition, it is proposed to add a complementary filter to a smart PID (Proportional + Integral + Derivative) controller reported in [44]. Therefore, experimental results show that the use of the complementary filter to the output signals of inertial measurement unit (IMU) helps to improve the performance of the smart PID controller used for the trajectory control, allowing to capture better quality of underwater images. Furthermore, experimental results show that the proposed ROV can process up to 42 frames per second for underwater video capture, due to its capability of performing parallel processing taking advantage of the threading library.

The rest of this paper is organized as follows: Section 2 describes the development, hardware organization, complementary filter, smart PID controller, and mechanical design of the proposed ROV. Section 3 describes the main algorithms performed by the computer onboard the ROV: (i) remote communication; (ii) motor control and temperature measurement; (iii) 3D position sensing; and (iv) video capture in real-time. Section 4 presents the experimental results, such as ROV performance, some images captured in real-world underwater environments, and a comparison versus commercial ROVs. Finally, Section 5 summarizes the conclusions of the paper.

## 2. Design and Construction of the ROV

### 2.1. Electronic Design

**Figure 1:**
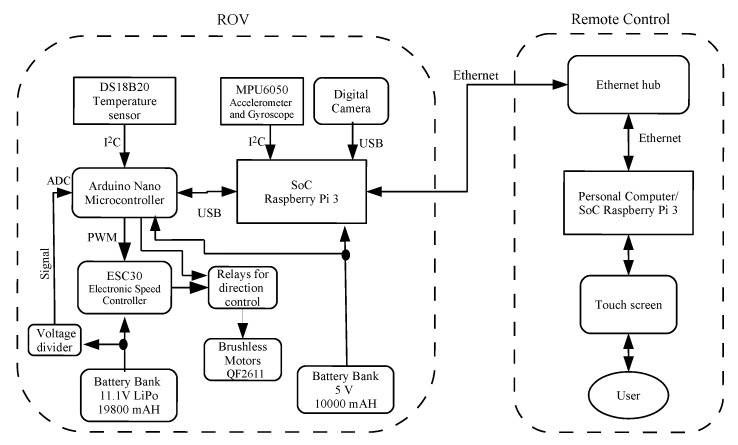
Block diagram of the proposed ROV.

Figure 1 shows the block diagram of the proposed hardware that consists of two parts: the remotely controlled vehicle and the remote control. These two subsystems are communicated through an Ethernet cable with Kevlar reinforcement, so that the user can control the ROV from a personal computer, laptop or SoC, all with Virtual Network Computing (VNC) connectivity. The ROV hardware involves a SoC Raspberry Pi 3 that is responsible for executing parallel tasks, such as: (i) acquisition of video by means of a digital camera; (ii) measurement and recording of the different variables associated with the sensors of the system; (iii) motors control in coordination with an Arduino Nano microcontroller; (iv) battery voltage monitoring for energy consumption and internal temperature; and (v) communication management with the remote control. The used digital camera is a Vemont full HD 1080 p of 12 Megapixels, which is built-in with an IP68 waterproof case, *allowing to dive underwater*. Besides, two power sources are integrated into the system, one with a power bank of 5 V with 10,000 mAh for the Raspberry Pi 3, an Arduino Nano microcontroller, and digital sensors, and the other one is a bank of six batteries of 11.1 V with 19,800 mAh to power six brushless motors. With these battery banks, the ROV has an autonomy of up to 2 h. The remote control shown in Figure 1 consists of an Ethernet network hub (Ethernet switch/router) interconnected by an Ethernet network cable to a computer or SoC, from which the user controls the ROV through a touchscreen type graphical user interface (GUI).

The brushless motors are model QF-2611 manufactured by XCSOURCE with a turning speed of 4500 KV three-phase voltage, are connected in a star configuration. The brushless motors are controlled by an electronic speed controller (ESC), which generates a three-phase output signal, having as an input a pulse width modulation (PWM) signal generated by an Arduino Nano microcontroller. Based on the experiments, it was found that 1 μs is the most efficient time to provide the best ROV stability underwater for video capture. The ESC30A delivers up to 30 A.

**Figure 2:**
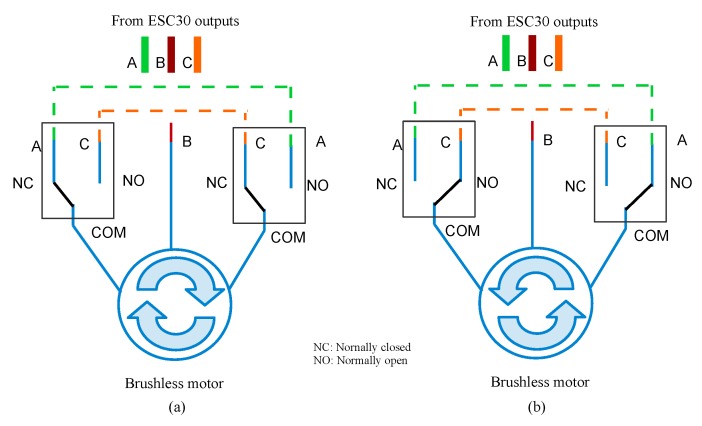
Electric circuit to invert the rotation direction of the motors: (**a**) turning the motor clockwise; and (**b**) turning the motor counterclockwise.

The speed controllers are unidirectional, and the changes in their rotation directions are shown in Figure 2. The circuit includes a pair of relays per motor, so that two phases reach the motor’s terminals, generating the change in the direction of rotation of the ROV according to the operation requirements.

Regarding Figure 1, the ROV incorporates the digital sensor DS18B20 for measuring the temperature inside its capsule, which is communicated through the I${}^{2}$C protocol directly to the Arduino Nano microcontroller.

**Figure 3:**
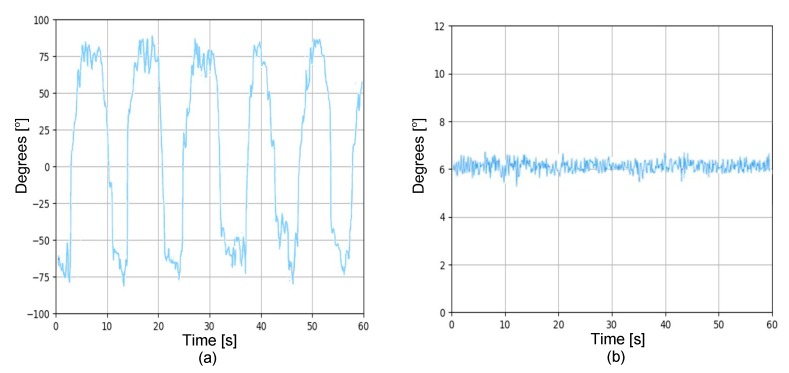
Output signal of the MPU6050 sensor without conditioning: (**a**) ROV in motion with direction in *y*; and (**b**) ROV without movements (parked).

**Figure 4:**
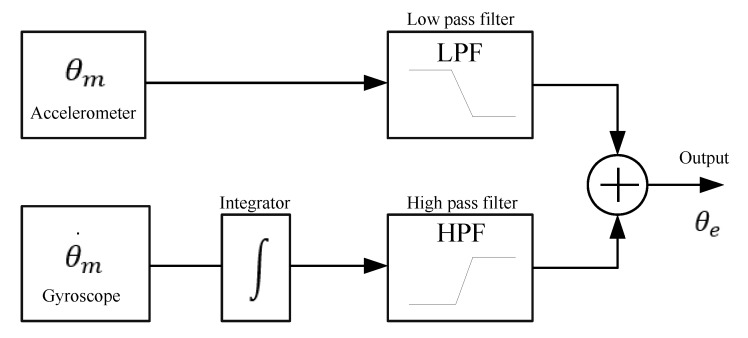
Scheme of the complementary filter [45,46,47] implemented on the ROV.

Due to the fact that at several meters underwater there is no line of sight between the remote control and the ROV, it is necessary to measure the precise position and orientation. That is why, knowing the 3D angular position of the ROV serves to automatically control its movement. This is done by using an MPU6050 sensor, which has six degrees of freedom. This means that there are three accelerometers and three gyroscopes inside the MPU6050. Figure 3 shows the measurements of one of the signals achieved directly at the output of the MPU6050. It can be appreciated that in both conditions of movement and static there exists the presence of small variations (noise vibrations), i.e., the forces driving the ROV are also perceptible in the sensor, which affect the stability of the ROV’s movements. To reduce these variations, a complementary filter [45,46,47] is used to filter undesired effects and therefore the Raspberry Pi 3 is used to process the MPU6050 data. In the complementary filter, the cutoff-frequency of the low-pass filter is designed considering the accelerometer measurements, while for the high-pass filter, its design depends on the signal measured by the gyroscope. The  schematic of the complementary filter is shown in Figure 4.

By mixing data from the accelerometer and gyroscope, the next transfer function for the complementary filter is obtained [45,46,47],

(1) $$\theta_{e}\left(z\right)=\frac{\omega_{c1}}{\left(1-z^{-1}\right)/dt+\omega_{c1}}\theta_{m}\left(z\right)+\frac{dt}{1-z^{-1}}\cdot \frac{(1-z^{-1})/dt}{\left(1-z^{-1}\right)/dt+\omega_{c2}}{\dot{\theta }}_{m}\left(z\right),$$

where $\theta_{m}$ is the angular data measured with the accelerometer in degrees [${}^{\circ }$], ${\dot{\theta }}_{m}$ is the angular velocity data measured with the gyroscope [${}^{\circ }/$s], $\theta_{e}$ is the output of the complementary filter in degrees [${}^{\circ }$], $\omega_{c1}=5$ Hz is the cutoff frequency of the low pass filter, $\omega_{c2}=2$ Hz is the cutoff frequency of the high pass filter, and dt is the differential time [s]. By simplifying and rearranging the transfer function, the following is obtained:

(2) $$\theta_{e}\left(z\right)\cdot \left[\left(1-z^{-1}\right)/dt+\omega_{c}\right]=\omega_{c}\cdot \theta_{m}\left(z\right)+\dot{\theta_{m}}\left(z\right),$$

by applying the inverse Z transform,

(3) $$\theta_{e}\left(k\right)\cdot \left(1/dt+\omega_{c}\right)=\theta_{e}\left(k-1\right)/dt+\omega_{c}\cdot \theta_{m}\left(k\right)+\dot{\theta_{m}}\left(k\right),$$

therefore,

(4) $$\theta_{e}\left(k\right)=\left(1-\alpha \right)\cdot \left[\theta_{e}\left(k-1\right)+\dot{\theta_{m}}\left(k\right)\cdot dt\right]+\alpha \cdot \theta_{m}\left(k\right),$$

where α=0.02 and *k* is the sample time.

**Figure 5:**
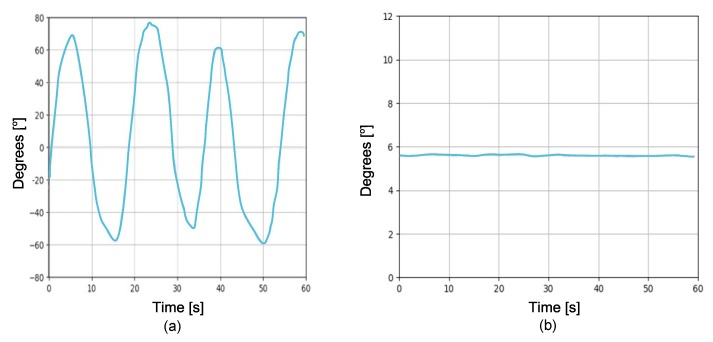
Motion sensor signal by using complementary filter: (**a**) ROV in motion with direction in *y*; and (**b**) ROV parked.

By implementing the complementary filter, greater ROV handling stability is obtained, as shown in Figure 5. It can be appreciated that, when the complementary filter is used, the ROV gains better stability, because the noise is significantly reduced. Hence, the above contributes in a quality improvement of captured images.

#### Control and Actuation Subsystems

It is well known that ROVs are subject to parametric changes (weight, buoyancy, added mass, payload, etc.) or external disturbances such as ocean currents; thus, the control subsystem used in the proposed ROV is inspired by the smart PID controller reported by Hernández-Alvarado et al. [44], which has the advantage of online tuning the gains of the PID controller as it is based on an artificial neural network (ANN). However, in this paper, it is proposed to add a complementary filter on the MPU6050 sensor output to improve PID controller performance and ROV stability, with the purpose of improving the quality of underwater images.

**Figure 6:**
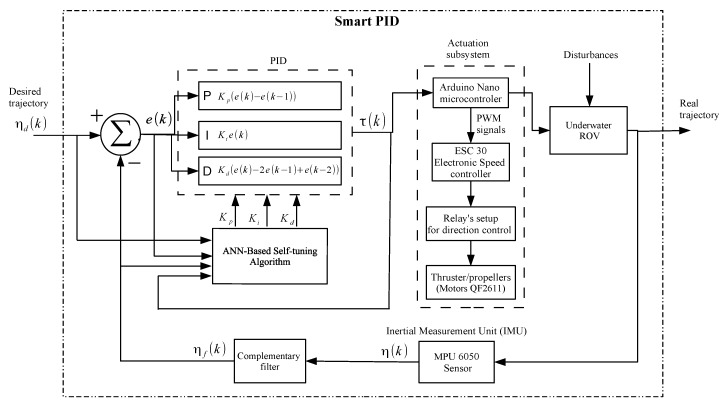
Block diagram of smart PID controller with actuation subsystem and complementary filter.

In the domain of discrete time, the digital PID algorithm can be expressed as [44]:

(5) $$\tau \left(k\right)=\tau \left(k-1\right)+K_{p}\left(e\left(k\right)-e\left(k-1\right)\right)+K_{i}e\left(k\right)+K_{d}\left(e\left(k\right)-2e\left(k-1\right)+e\left(k-2\right)\right),$$

where τ(k) is the original control signal, $e\left(k\right)=\eta_{d}\left(k\right)-\eta_{f}\left(k\right)$ represents the position tracking error, $\eta_{d}\left(k\right)$ denotes the desired trajectory, η(k) is the real trajectory without processing, $\eta_{f}\left(k\right)$ are the filtered data of real trajectory, $K_{p}$ is the proportional gain, $K_{i}$ the integral gain, $K_{d}$ is the derivative gain, and *k* is the sample time. Figure 6, depicts the block diagram of self-tuning PID controller based on ANN [44]. It can be observed that the use of the complementary filter described in Figure 4  reduces noise levels at the output of the MPU 6050 sensor. The stage of the actuation subsystem, as depicted in Figure 1, is integrated with an Arduino Nano microcontroller, the power stage with six ESC30A speed controllers, twelve relays for controlling the rotation direction of thrusters (Figure 2), and six QF2611 thrusters/motors with propellers.

**Figure 7:**
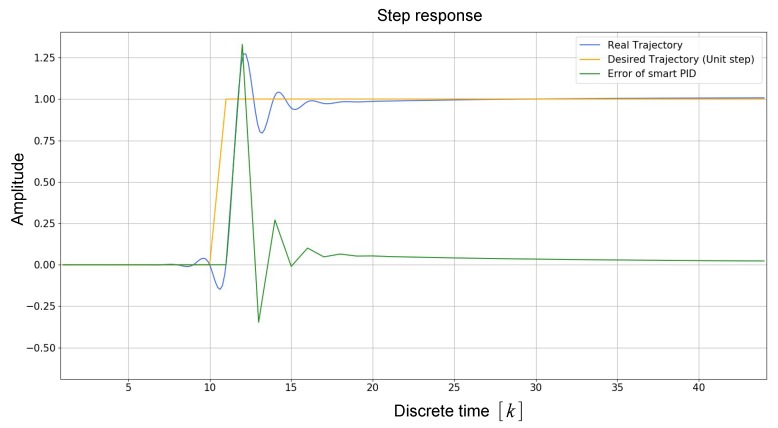
Simulation of smart PID controller’s response to unit step input.

Figure 7 shows a simulation of smart PID controller’s response to unit step input during a time lapse of 50 ms. It can be observed that, when an abrupt change is produced in the input, its stabilization response time is less than 10 ms.

### 2.2. Mechanical Design

The physical characteristics of materials that were used in designing the ROV to operate in underwater conditions can be seen in [48]. In this work, polyvinyl chloride (PVC pipe with steel bars inside) is used as a structural element since it complies with mechanical characteristics for the ROV to function properly, and determines the maximum depth that can be reached. The depth is calculated by Equation (6); it requires knowing the fracture point of the material provided by the manufacturer, which in this case corresponds to a pressure of 1724 kPa. Under these conditions, the maximum depth for a PVC pipe with schedule 40 is h=125.65 m.

**Figure 8:**
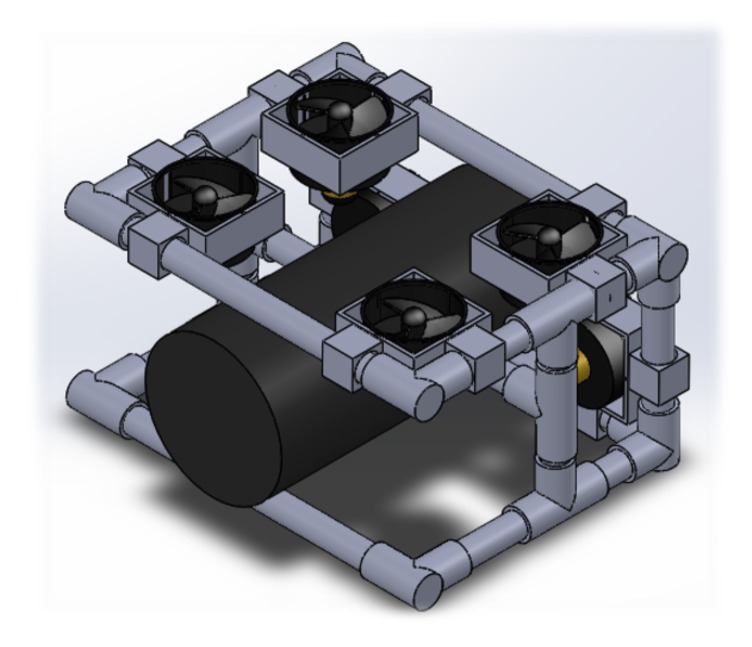
3D design view of the proposed ROV.

(6) P=ρ·g·h,

where *P* is pressure, ρ=1400 kg/m${}^{3}$ is seawater density, *g* is gravity, and *h* is depth in meters. The 3D structural design of the proposed ROV is shown in Figure 8. The design takes into account aspects of hydrostatic and buoyancy depending on the application environment, either a marine environment (saltwater) or under controlled conditions (fresh water). The structure presented considers a weight balance configuration to facilitate the implementation in the topology of the motors, and it was developed in the 3D figure simulator software known as SolidWorks. Its physical dimensions are 18.41 cm × 29.50 cm × 33.50 cm, with an estimated volume of $V=18.19\times 10^{-3}$ m${}^{3}$ and an estimated weight of W=15.64 kg. Based on these data and considering a saltwater density of ρ=1400 kg/m${}^{3}$, the buoyancy corresponds to 249.6 N, which can be obtained by Equation (7), where *E* is the total thrust, ρ is the density of the fluid, *g* is the gravity, and  *V* is the volume. Therefore, the proposed topology for the six motors in the ROV considers a minimum thrust force of 9.82 kg.

**Figure 9:**
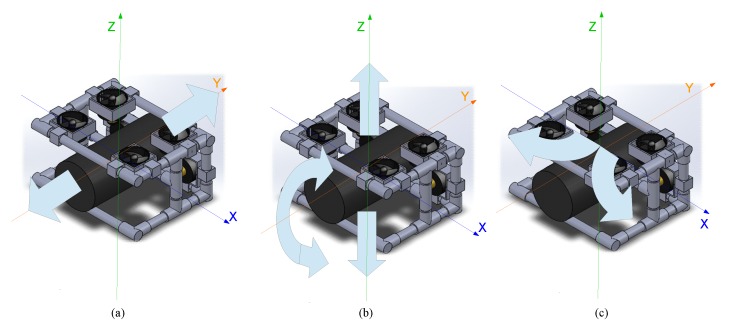
Movements of the ROV: (**a**) translational; (**b**) ascent and descent; and (**c**) rotation.

(7) E=−ρ·g·V,

Figure 9 shows the locations of the six brushless motors: four are located on the top for the ascent and descent movements and two on the front for translational and rotational movements.

According to Figure 8, Table 1 shows the materials used in the construction of the mechanical structure of the ROV. The designed mechanical structure allows the placement of the six brushless motors, as can be seen in Figure 9. Table 2 shows the components with their estimated weights that give the total weight of 15.64 kg. To achieve mechanical stability within the water and operate with the buoyancy estimated by Equation (7), eight steel bars are incorporated into the structure. The final design of the ROV structure allows placing the camera in different positions, i.e.  it can be placed in front, under, or on the sides of the ROV.

## 3. ROV Algorithms

**Figure 10:**
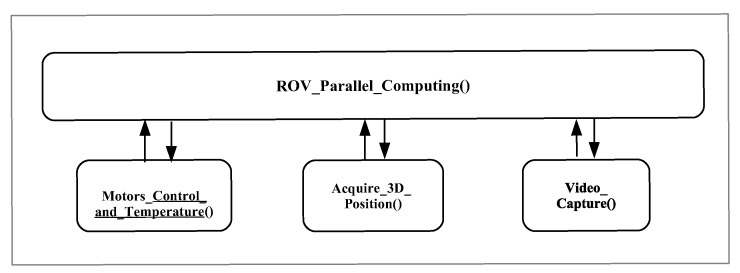
Algorithms executed on the Raspberry Pi 3 performing parallel computing.

Figure 10 shows the main algorithms executed by the SoC Raspberry Pi 3 that is used as the ROV’s onboard computer, which are divided into four parts: (i) main control of the whole structure and communication (**ROV_Parallel_computing**); (ii) motors control and temperature measurement **(Motors_Control_and_Temperature)**; (iii) 3D position sensing **(Acquire_3D_Position)**; and (iv) video capture **(Video_capture)**. For the programming and execution of these algorithms, Python software version 2.7.9 was used.

**Figure 11:**
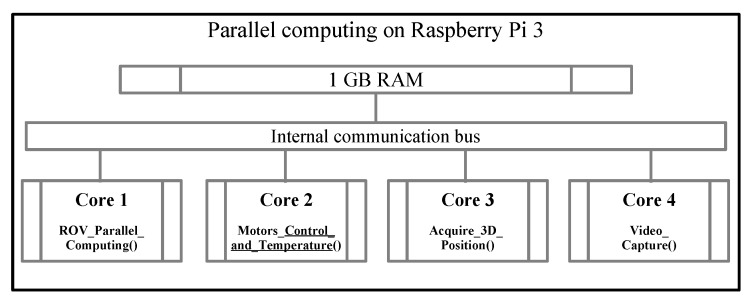
Block Diagram of multicore SoC Raspberry Pi 3.

Furthermore, the Raspberry Pi 3 SoC contains four cores inside its microprocessor with a 1 GHz clock, so each algorithm is mapped to each core for execution. Figure 11 depicts an outline of the multicore organization. The communication between the ROV and the remote control is via Ethernet and can be controlled remotely with different computer hardware or SoC, allowing the use of different operating systems in the computer connected to the Ethernet network hub, as described in Figure 1. The Raspberry Pi 3 works with the GNU/Linux operating system Raspbian distribution, and the remote computer/SoC works using the VNC protocol, which is compatible with Windows, Android, OS X, or GNU/Linux.

The code fragment shown in Algorithm 1 describes the main algorithms executed on the SoC Raspberry Pi 3. On Line 1–5, the sys, Tkinter, cv2, threading, and serial libraries are included. The sys library is included in the ROV application; commands from the GNU/Linux operating system can be executed. The Tkinter Library is used for the development of graphical user interface. The cv2 library contains subroutines for image processing, such as acquisition, display, etc. The threading library is used to execute the algorithms shown in Figure 10 in parallel. The serial library is used to manage the serial communication between Raspberry Pi and the Arduino Nano microcontroller. On Line 6, the main function **ROV_Parallel_Computing()** is declared. On lines 7–25, the function **Motors_Control_and_Temperature()** is declared, which is used to control the motors for the movement of the ROV by using the smart PID controller, as depicted in Figure 6, and to measure the internal temperature by using the DS18B20 sensor. On lines 26–30, the function **Acquire_3D_Position()** is declared, which is used to check the 3D position of the ROV by means of the MPU 6050 sensor. On Lines 31–45, the **Video_Capture()** function is declared, which is used to capture video in real time through the camera. On Lines 46–52, the code is used to setup the serial communication between Raspberry Pi and the Arduino Nano microcontroller. On Lines 53–55, the program sends a message in the case of a connection error and to exit the ROV application. Finally, Lines 57–64 execute the ROV’s algorithms by using parallelism with threads. The function **Motors_Control_and_Temperature()** controls the movements required by the ROV via the smart PID controller, such as those shown in Figure 6 and Figure 9, which are carried out by an embedded algorithm developed for the Raspberry Pi 3, and it communicates with the Arduino Nano microcontroller to generate the PWM signals that will control the motors’s speed, which is composed of four algorithms: (i) calibration of the speed controllers; (ii) adjustment of the direction of rotation through relays; (iii) speed adjustment through a signal using pulse width modulation (PWM) with steps of 1 μs; and (iv) motor control via the smart PID controller. This function also measures the capsule’s interior temperature where the ROV electronics are located. Regarding the function **Acquire_3D_Position()**, the acquisition of the ROV position is performed by the SoC Raspberry Pi, providing information on the acceleration about inclination angles and angular velocity data. It also provides six-axis digital information, uses communication with I${}^{2}$C protocol, and operates at 3.3 V. The code fragment presents the three algorithms of execution: (i) declaration of the communication port; (ii) acquisition of data from the acceleration and rotation; and (iii) angle filtering.

Since the algorithms are programmed using open software tools, the whole system allows the addition of more sensors and functionalities to the ROV, according to the operational needs, maintenance, supervision, and sense of physical-chemical variables underwater.

**Algorithm 1** Executing the ROV’s algorithms by performing parallel computing.
1:import sys                     # System parameters and functions2:from Tkinter import  # Library for GUI3:import cv2                    # Library for image processing  4:import threading       # Library for parallel computing by threats  5:import serial                  # Library for serial communication  6:**def ROV_Parallel_Computing ( )**  7:     **def Motors_Control_and_Temperature( )**  # Function for motor control and temperature sensing.  8:        switch( PushButton ( ) ) # Configuration of the motions  9:            case 1:  10:               def Config_UP ( )  11:            case 2:  12:               def Config_DOWN ( )  13:            case 3:  14:               def Config_LEFT ( )  15:            case 4:  16:               def Config_RIGHT ( )  17:            case 5:  18:               def Config_STOP ( )  19:            case 6:  20:               def Config_CALIBRATE ( )  21:         if(Serial.read == TEMP) # Read temperature  22:            Read Temperature  23:            Print Temperature.  24:            Store Temperature.  25:         endif  26:     **def Acquire3D_Position( )**    # Read 3D position  27:          def Read_Acl( )       # Read the accelerometer data  28:          def Read_Gyr( )       # Read the gyroscope data  29:          def filter_Comp( )   # Filtering accelerometer and gyro.  30:          print( ’positon’ )  31:     **def Video_Capture( ):**  32:          print ("Camera")  33:          cap=cv2.VideoCapture(0)       # Video capture.  34:          while (1)  35:              ret, FPS = cap.read().  36:              cv2.imshow(’ROV CAM’,frame)  37:              CPUTEMP = Read_CPU_temp.  38:              print (FPS, CPUTEMP)  39:              key= cv2.waitKey(1)  40:              if key == 27:  41:                     break  42:          endwhile  43:          cap.release.  44:          cv2.destroyallWindows.  45:     ROV_Parallel_Computing.mainloop()  46:**try:**         # Setup communication with Arduino Nano Microcontroller.  47:                   ard = serial.Serial()  48:                   ard.port = ’/dev/ttyUSB0’.  49:                   ard.baudrate = 9600.  50:                   ard.timeout = 3.  51:                   ard.open().  52:                   print(’Wait a moment....’).  53:**except:**.  54:                   print(’Connection Error’)  55:                   sys.exit().  56:# **Parallel algorithms execution**.  57:# Assign a thread for parallel execution.  58:CAMERA=threading.Thread(target = Video_Capture )                     59:CAMERA.start().  .60:POSITION=threading.Thread(target = Acquire_3D_Position )              61:POSITION.start().  62:MOTORS=threading.Thread(target = Motors_Control_and_Temperature )  63:MOTORS.start().  .64:APP=threading.Thread(target = ROV_Parallel_Computing)                 65:APP.start()  


## 4. Experimental Results

The experimental results using the Raspberry Pi 3 as the ROV’s onboard computer are summarized herein, as well as the hardware resources used by each algorithm and other processes running simultaneously. The captured images in a controlled aquatic environment, as well as obtained images in the sea environment are also shown. In addition, the results obtained in the implementation of the smart PID controller and a comparison of the main features versus two commercial ROV are presented.

### 4.1. ROV Performance

#### 4.1.1. Motors Test

**Figure 12:**
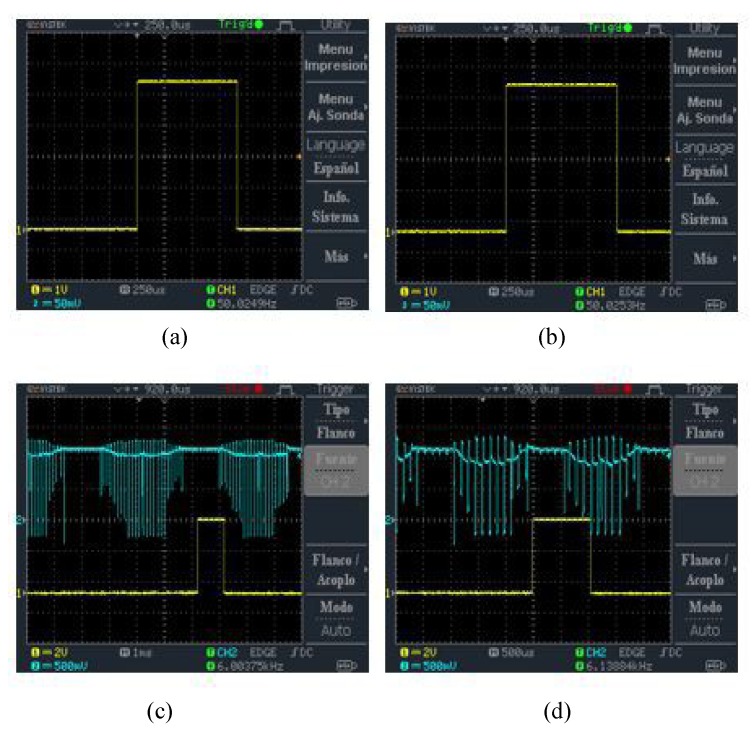
PWM signals generated by the Arduino Nano microcontroller: (**a**) PWM signal of average speed; (**b**) PWM signal of maximum speed; (**c**) PWM signal of minimum speed and output signal of ESC30A controller; and (**d**) PWM signal of maximum speed and output signal of ESC30A controller.

The Arduino Nano microcontroller and Ardu-Pilot development boards were used for the brushless motors speed and operation tests to identify which one is more efficient for the generation of the PWM signals required by the ESC30A. We found that the change made by the Ardu-Pilot flight controller was quite sudden for the brushless motors used in this paper. Due to this limitation, a program was implemented on the Arduino Nano microcontroller to generate higher resolution PWM signals for the speed changes. Based on the experimentation, it was found that 1 μs is the most efficient time and that it provides the best ROV stability under water. Figure 12 shows an example of PWM signals generated by the Arduino Nano microcontroller for the control of brushless motors. Figure 12a depicts the PWM signal of average speed, Figure 12b shows the PWM signal of maximum speed, Figure 12c depicts the PWM signal of minimum speed and the corresponding output signal of ESC30A controller, and Figure 12d depicts the PWM signal of maximum speed and output signal of ESC30A controller.

#### 4.1.2. Temperature in SoC Raspberry Pi 3 and ROV Capsule

**Figure 13:**
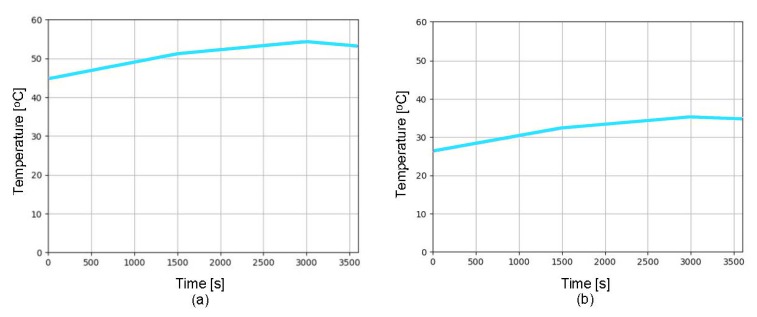
Temperature measurement of every second over a period of one hour: (**a**) temperature of the chipset Raspberry Pi 3; and (**b**) temperature in the ROV capsule.

Figure 1 shows that the ROV incorporates the DS18B20 digital sensor for measuring the temperature inside the capsule, which is communicated through the I${}^{2}$C protocol directly to the Arduino Nano microcontroller. An experimental behavior analysis of the internal temperature in the ROV capsule was required due to the heating produced by the ESC30 when operating the motors. For these measurements, an embedded algorithm on the Arduino Nano microcontroller was employed, obtaining temperature data from the DS18B20 sensor. Additionally, an algorithm was implemented in the SoC Raspberry Pi 3 to measure the temperature of its chipset, given that the maximum operating temperature must not exceed 65 ${}^{\circ }$C. Figure 13a shows the temperature behavior from the Raspberry Pi 3 chipset. Figure 13b shows the temperature inside the ROV capsule. The figure shows that, when the motors are turned on at their maximum speed, the temperature of the Raspberry Pi3 chipset increases, reaching 54 ${}^{\circ }$C in less than 1 h, while the temperature of the ROV capsule rises up to 37 ${}^{\circ }$C. On the other hand, when the motors are turned off, the chipset and capsule temperature decrease over time.

#### 4.1.3. Battery Banks Performance

**Figure 14:**
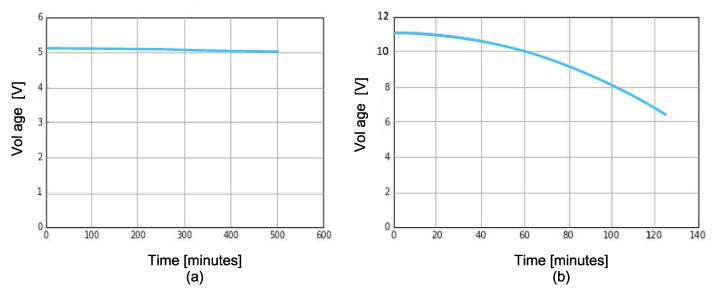
Behavior of the 5 V and 11.1 V battery banks during the operation of the ROV: (**a**) 5 V with 10,000 mAh battery bank for digital devices; and (**b**) 11.1 V with 19,800 mAh battery bank for brushless motors.

The performance of the battery banks under ROV operation is shown in Figure 14. The behavior of the 5 V battery bank supplying power to the digital components is shown in Figure 14a, where it is observed that it has a work time of 500 min (8.33 h) continuous, executing the algorithms that control the ROV. Meanwhile, the battery bank of 11.1 V, which energizes the ESC30A for the control of the brushless motors has a time duration of 120 min (2 h) at medium speed, as shown in Figure 14b. However, this time may increase or decrease according to the navigation of the ROV.

#### 4.1.4. Stability Performance

**Figure 15:**
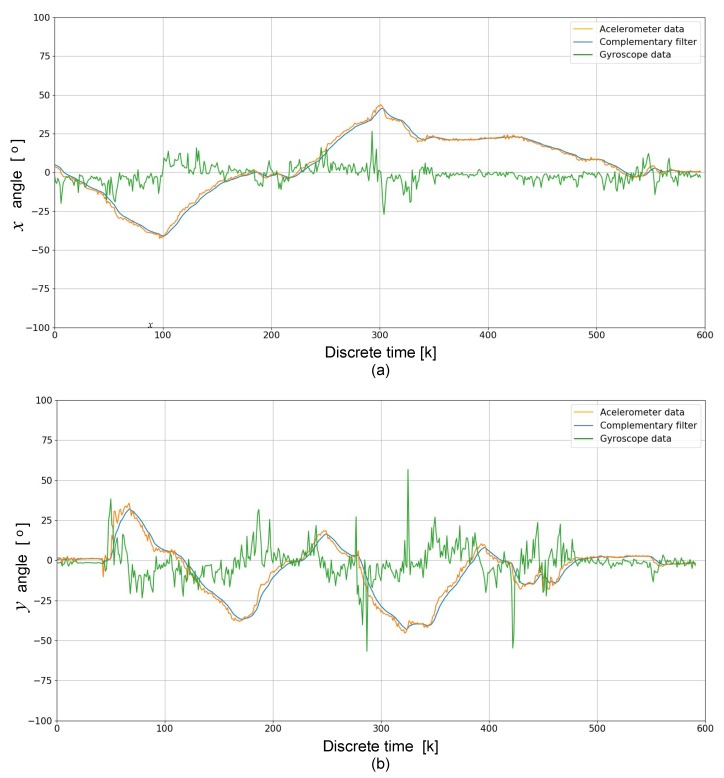
Stability tests of the ROV on the output of the complementary filter: (**a**) movements on *x*-axis; and (**b**) movements on *y*-axis.

The MPU6050 sensor is responsible for colorredkeeping track of the orientation of the ROV and is very useful to stabilize it. The complementary filter gives us a smoothed signal from both signals (accelerometer and gyroscope). In the short term, we used the data from the gyroscope, because they were very precise and not susceptible to external forces. In the long term, we used the data from the accelerometer, as they did not drift. In this experiment, the used sensitivity for the accelerometer was 16,384 LSB/*g* and for the gyroscope was 131${}^{\circ }/$s. Figure 15 depicts the stability tests on the output of the complementary filter. Figure 15a shows movements on *x*-axis, while Figure 15b shows movements on *y*-axis. It can be observed that no noisy accelerometer and gyroscope data were detected, i.e., they did not drift away. In addition, the complementary filter helped improve the stability of the ROV and the quality of the captured images was improved. Finally, the complementary filter is easy and light to implement on embedded systems such as Raspberry Pi.

**Figure 16:**
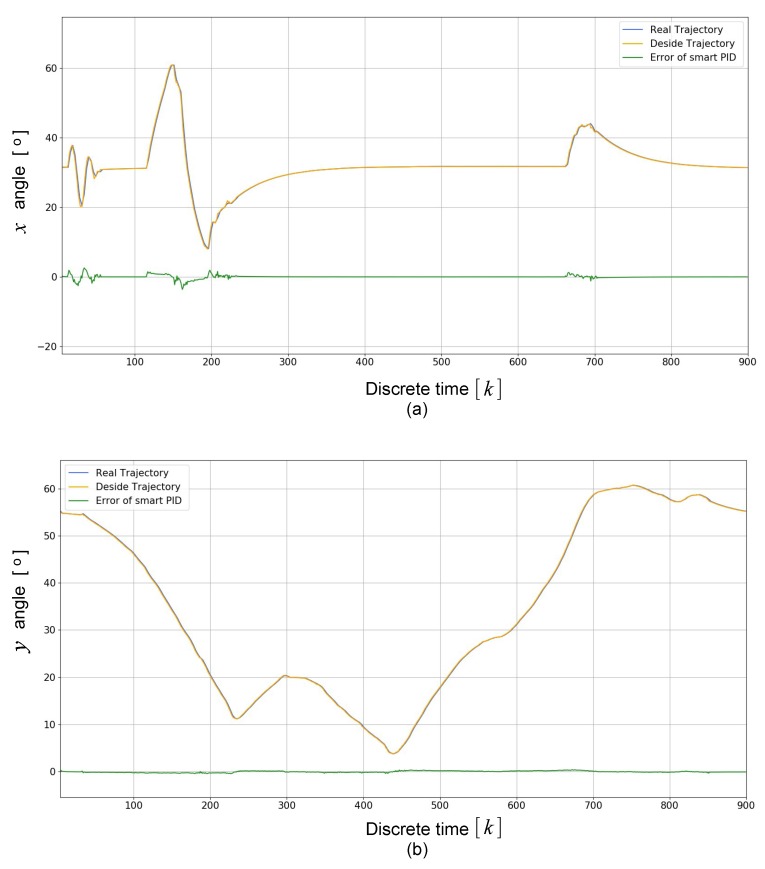
Smart PID controller’s response: (**a**) *x*-direction; and (**b**) *y*-direction

Figure 16 depicts the smart PID controller’s response. Figure 16a,b shows the angle in *x*-direction and *y*-direction, respectively. It can be observed that the real trajectory followed the desired trajectory. In the control of both trajectories, it is appreciated that the error was close to zero, and the signals were smoothed as a result of the implementation of the complementary filter. Therefore, this helps to improve the stability of the ROV and consequently the quality of the images captured underwater.

### 4.2. Hardware Resources

**Figure 17:**
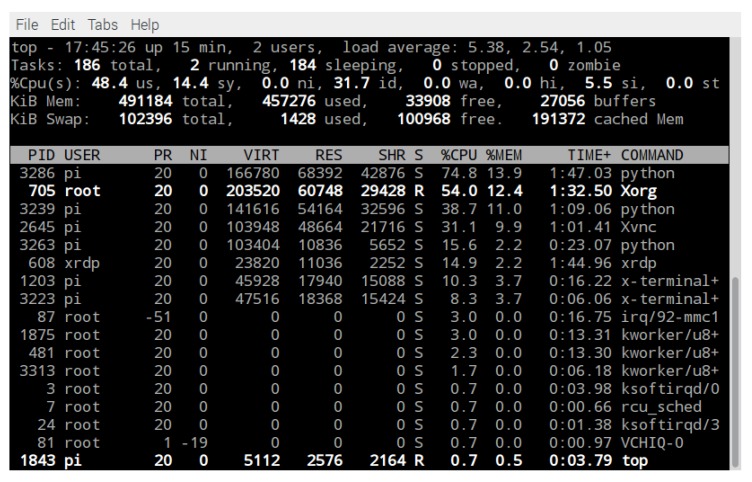
Hardware resources in the Raspberry Pi 3.

Figure 17 shows the used resources by all the algorithms and other processes running on Raspberry Pi 3 SoC as the onboard computer of the ROV. It can be observed that the process labeled PID 3286, in the left column, which corresponds to the main algorithm labeled as ROV_Parallel_Computing in Figure 10, used 74.8% of the CPU resources, and 13.9% of RAM memory. The PID 3239 that corresponds to Video_Capture algorithm used 38.7% of the CPU resources and 11.0% of RAM memory. The PID 2645 corresponding to the VNC protocol used 31.1 % of the CPU resources and 9.9 % of RAM memory. The PID 3263 corresponding to the Acquired_3D_Position algorithm that receives the data from the position sensor MPU 6050 and graphs the 3D position of the ROV used 15.6% of the CPU resources and 2.2% of RAM memory. The PID 1203, which manages the motor control algorithm, labeled as Motors_Control in Figure 10, used 10.3% of the CPU resources and 3.7% of RAM memory. Finally, the PID 3223 corresponding to the Measure_Temperature algorithm, associated to the sensor DS18B20, used 8.3% of the CPU resources and 3.7% of RAM memory.

**Figure 18:**
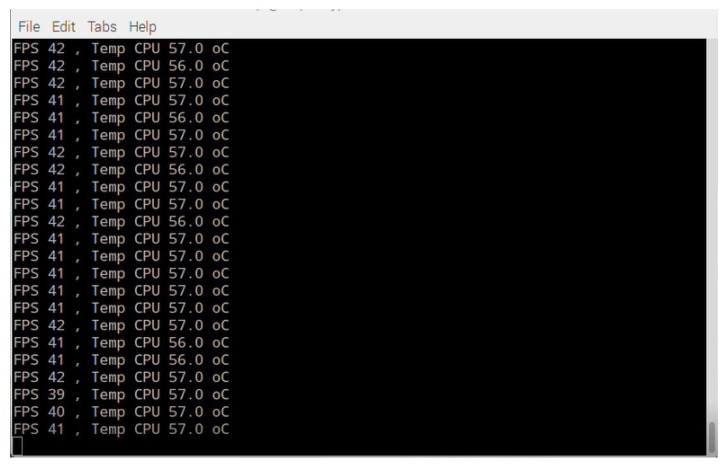
Experimental results of the number of captured frames per second (FPS), and temperature measurement provided by the Raspberry Pi 3.

Figure 18 shows the experimental results of the images captured by the camera given in frames per second (FPS), which are processed by the Raspberry Pi 3. It can be appreciated that the maximum speed of video capture is 42 FPS.

### 4.3. Tests in Controlled Aquatic Environment

**Figure 19:**
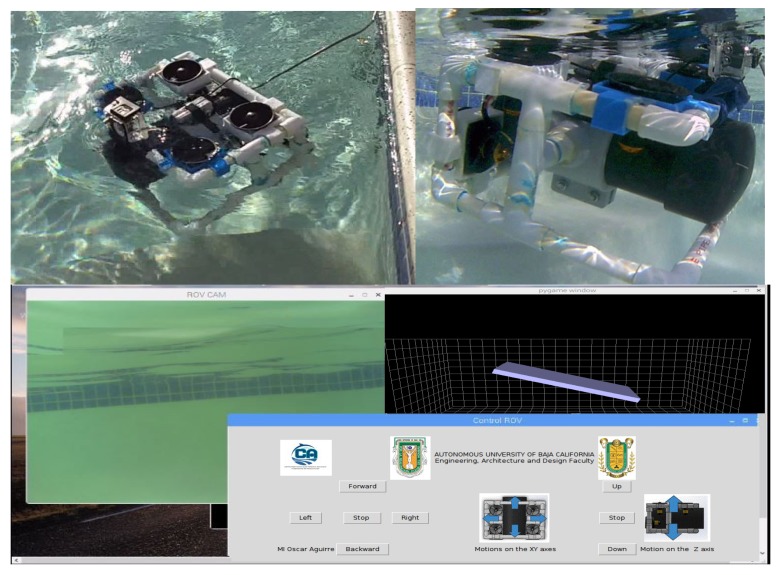
Images captured in a controlled aquatic environment.

Figure 19 depicts the captured images by the ROV camera in a controlled aquatic environment. These tests also verified that the water does not leak inside the ROV capsule, and proved its buoyancy in the water. The top of Figure 19 shows the submerged ROV in the aquatic environment. The bottom of Figure 19 depicts an underwater image captured by the ROV. The remote control interface of the ROV can also be observed, which could work using a touch screen. In addition, a 3D graphic indicates the ROV position.

### 4.4. Tests in Real-World Scenario

**Figure 20:**
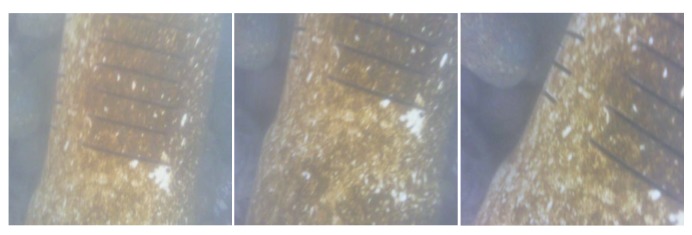
Inspection test images of a suction valve in subsea water intake.

**Figure 21:**
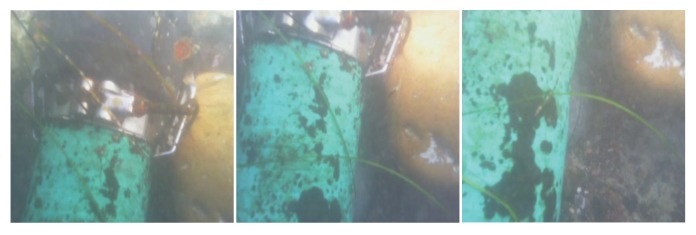
Underwater images of PVC pipe fasteners.

**Figure 22:**
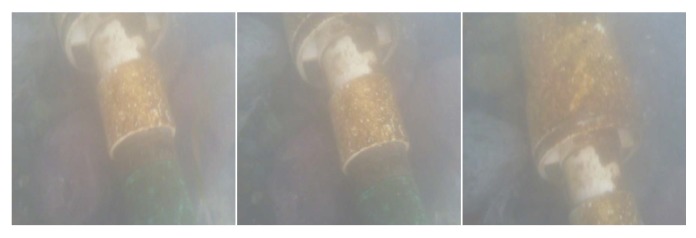
Underwater images of current status of connections between PVC pipes.

**Figure 23:**
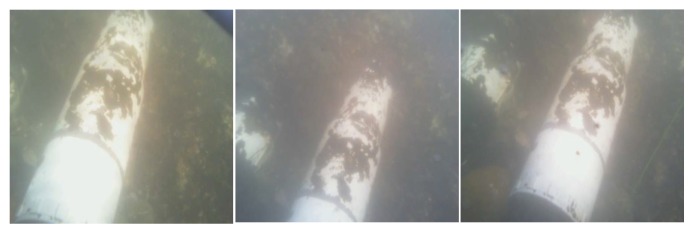
Images of connections between PVC pipes placed underwater.

**Figure 24:**
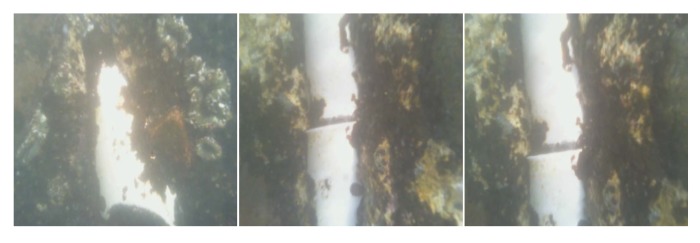
Images of incrustations on underwater PVC pipes.

**Figure 25:**
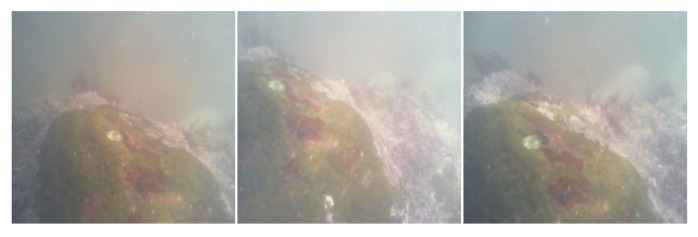
Images under seabed conditions.

This section presents different images obtained by the ROV in a submarine environment. To carry out these tests, the camera was placed at the lower part of the ROV; this so that the images of the PVC pipe installed on the seabed could be captured. The images were taken at the seawater suction stage, located on the coast at 2.4 m depth. This seawater was then processed by several filters, used to feed various fish and mollusk culture ponds, at a biotechnology area for aquaculture. As an example, several image sequences are shown in groups of three, which allows the visualization of the potential of the proposed ROV for various applications and environments. As shown in Figure 15, the ROV captured video of up to 42 FPS, and from these sequences images can be extracted, as shown in Figure 20, Figure 21, Figure 22, Figure 23, Figure 24 and Figure 25. In all these cases, one can observe three frames that were obtained in a real-world scenario, such as the subsea, where water turbidity is noticeable.

Figure 20 depicts a sequence of a valve for suction in a subsea water intake. Different approaches can be observed and the external condition of the valve, a sediment film in its exterior, is particularly distinguished. This type of inspection is of great value, because, if the valve inlets were clogged, it could cause a greater deterioration to the suction pumps. Furthermore, due to excess of sediment, the water’s flow through the suction valve might become blocked, which could cause greater damage to the suction pump and water shortage for the aquaculture ponds.

Figure 21 and Figure 22 depict two sequences of images from different sections of a PVC pipe used for seawater suction. Different approaches and the external conditions of the pipe are observed. In addition, different types of connections and the conditions in which they are found are highlighted here. This type of visual inspection, obtained remotely with the ROV, serves to detect any physical damage to the pipe or connections, which is important in preventive maintenance routines for this type of installation.

Figure 23 and Figure 24 depict two sequences of images of a PVC pipe. Different approaches are shown; they specifically highlight the incrustations that have formed around them over time. This inspection is of great value as it make the conditions in which the pipe is found evident and the latent risk that this can generate throughout the system, such as a leak of some sort of substance.

Figure 25 shows a sequence of three images of a seabed region. Different approaches of its topography are observed. This type of inspection is of great value, for example for the study of fauna, flora, and for the search of microorganisms underwater.

### 4.5. Comparison with Other ROVs

Table 3 shows a features comparison of proposed ROV versus commercial ROVs. It can be observed that some features are the same. However, the proposed ROV has parallel computing capacity, which allows simultaneously executing several tasks or processes and capturing images of up to 42 FPS with a resolution of 800 × 640 pixels. The implementation of the complementary filter (see Figure 4) to the IMU signals (see Figure 15) helps to improve the performance of the smart PID controller (see Figure 6) used for the trajectory control (see Figure 16), allowing to capture better quality of underwater images (see Figure 20, Figure 21, Figure 22, Figure 23, Figure 24 and Figure 25). The remote control of the ROV is executed under a graphical user interface coded on Python, which is suitable for different operating systems, such as GNU/Linux, Windows, Android, and OS X. The payload is the carrying capacity of a ROV, e.g., to add more sensors, measuring instruments, samples collected from the ocean, etc. The payload of the proposed ROV is 20% of its total weight, i.e., 3.128 kg. On the other hand, given the characteristics of the proposed mechanical and hardware design, the maintenance and replacement of mechanical and hardware components allows technological independence of ROV manufacturers.

## 5. Conclusions

The ROV development and implementation contained in a box of size 18.41 cm × 29.50 cm × 33.50 cm, with a weight of 15.64 kg, is introduced. The proposed ROV performs the translational, ascent, descent, and rotational movements on three axes to capture images of 800 × 640 pixels on video graphic array standard. The ROV design was done to reach up to 100 m underwater, which can solve the problem of divers who can only reach 30 m. The motion control, 3D position, temperature sensing, and video capture are performed in parallel by using the threading library, and they are processed by a main algorithm that was programmed to use the four cores of a SoC Raspberry Pi 3. The communication between the ROV and the remote control is handled by a graphical user interface coded on python, which is suitable for different operating systems, such as GNU/Linux, Windows, Android, and OS X. Furthermore, the ROV moves under the six brushless motors governed through a smart PID controller. From experimental results, the brushless motors were calibrated to work with a short pulse width of 1 μs to improve the ROV stability and underwater position. A complementary filter used to smooth noise vibrations from the MPU 6050 sensor improves ROV stability. Therefore, it helps to improve the captured video quality by processing up to 42 FPS on a Raspberry Pi 3. The autonomy of the proposed ROV is up to 2 or 3 h. The algorithms were programmed using open software tools, which allows adding more sensors and functionalities, according to the needs of operation, maintenance, supervision, and sensing of physical-chemical variables underwater. In addition, the flexibility of mechanical design and low-cost hardware increases potential applications, such as surveillance, fishing operations, growth control of fish, and study of marine flora and fauna, without demeriting the quality in the acquisition of the information, within a context of technological independence.

## Figures and Tables

**Table 1:** Materials for the construction of the ROV structure.

Quantity (Pieces)	Description	Size [cm]
4	PVC pipe	ϕ1.27 × 25
4	Corner connector	ϕ1.27
2	PVC pipe	ϕ1.27 × 11.5
5	T-connector	ϕ1.27
4	PVC pipe	ϕ1.27 × 5
4	L-connector	ϕ1.27
1	PVC pipe	ϕ10.16 × 25

**Table 2:** Weights of the ROV’s materials.

Item	Quantity	Weight [kg]	Total Weight [kg]
PVC structure	1	6.940	6.940
Battery 11.1 V	6	0.200	1.200
Battery 5.0 V	1	0.200	0.200
Raspberry Pi	1	0.100	0.100
Arduino Nano	1	0.001	0.010
Relays	6	0.150	0.900
ESC30A	6	0.050	0.300
Brusless motor	6	0.225	1.350
Steel bars	8	0.580	4.640
		**Total weight**	15.64

**Table 3:** Comparison of characteristics of the proposed ROV vs. commercial ROV.

Features	Blue ROV (Standard)	OpenROV	Proposed ROV
Architecture	Open	Open	Open
Camera 1080 p	Yes	Yes	Yes
Autonomy time [h]	2–3	2–3	2–3
Communication	Ethernet	Ethernet	Ethernet
Internet connectivity	Yes	Yes	Yes
Maximum depth [m]	100	100	100
Processing type	Unknown	Unknown	Parallel
Frames per second [FPS]	30	30	42
Controller algorithm	PID	PID	Smart PID
Remote control	Joystick	Joystick for	Graphic user
		Android 5	interface *
Payload [kg]	2.200	1.000	3.128
Dimensions (length×width×height) [cm]	45.7 × 33.8 × 25.4	8 × 20 × 40	18.4 × 29.5 × 33.5
Total weight [kg]	11.00	2.90	15.64
Total cost [USD]	2784.0	1695.0	600.0

* Multi-platform (Windows, Linux, Unix, Android).
